# Establishment and Characterization of a Highly Metastatic Ovarian Cancer Cell Line

**DOI:** 10.1155/2018/3972534

**Published:** 2018-06-25

**Authors:** Jiang Ruibin, Cheng Guoping, Zheng Zhiguo, Ni Maowei, Wan Danying, Feng Jianguo, Gu Linhui

**Affiliations:** ^1^Cancer Research Institute, Zhejiang Cancer Hospital, Hangzhou, Zhejiang 310022, China; ^2^Key Laboratory of Diagnosis and Treatment Technology on Thoracic Oncology, Hangzhou, Zhejiang 310022, China

## Abstract

Ovarian cancer leads the worst prognosis among all types of gynecologic malignancies, and patients are often diagnosed at an advanced stage. Ovarian cancer also has a high rate of metastasis; however, the detailed mechanisms for ovarian cancer prone to metastasis remain unclear. In this study, we used continuous in vitro screening of the human ovarian cancer A2780 cell line to establish a cell line (A2780-M) which shows high invasiveness and motility. Compared to the parental cells, A2780-M cells express elevated protein levels of CD44, CD133, CD34, and *β*-catenin. A2780-M cells are also more resistant to chemotherapeutic agents SN-38 and Docetaxel. Thus, the A2780-M cell line is a new ovarian metastatic cancer cell line that expresses tumor stem cell surface markers and adhesion-related membrane proteins and is with higher motility and invasiveness.

## 1. Introduction

Ovarian cancer is with the highest mortality among gynecological cancers, and metastasis is the major cause to the worst prognosis[1]. The 5-year survival rate of ovarian cancer is about 40%, and over 200,000 new cases are reported every year worldwide[2, 3]. Patients are often diagnosed at advanced stages of the disease, and metastasis can be detected in ~30% of patients at the first diagnosis, while additional 30%–40% of patients will develop metastases in a few years after diagnosis and treatment [4]. The current therapeutic strategies are usually ineffective for ovarian cancer metastasis, and mortality is thus high for the patients that ovarian cancer has metastasized. Therefore, ovarian cancer remains to be one of the major clinical problems and life-threat for woman.

The investigations of the cellular and molecular signaling correlating to the metastasis of ovarian cancer may provide essential helps for understanding the underlying molecular mechanisms and for identifying new targets for clinical diagnosis and clinical managements. To this setting, however, lacking cell modals that share the same genetic background but differ with metastatic potentials has become the major hamper for basic research.

Lung metastasis is one of the major types for distant metastasis of ovarian cancer [5]. Several established human ovarian cancer cell lines, AO, CAOV-3, CAOV-4, OVCAR-3, and HO-8910PM, have been found to have high incidence of lung metastasis in animal models[6–8]. However, no comparable cell populations, or cell lines, have been established from lung metastatic tumors of these ovarian cancer cell lines yet.

In this study, we established lung metastatic cell line, A2780-M, from human ovarian cancer A2780 cells and characterized the cell surface markers and the biological behaviors of A2780-M cells in response to chemodrug treatment, with comparing to that of the parental A2780 cells.

## 2. Materials and Methods

### 2.1. Establishment of the Cell Line

#### 2.1.1. Establishment of the A2780-M Cell Subline with Transwell Invasion Assay

The A2780 cell line was from the Chinese Academy of Medical Sciences Cancer Hospital (Beijing,China). 5 ×  10^∧^4 cells log phase growing cells were collected and loaded onto the polycarbonate membrane of Corning Transwell incubation chamber (Cat. No. 3422; Corning, NY, USA) precoated with Matrigel (Cat. No. 356234; Corning). RPMI-1640 medium (GIBCO,NY,USA) supplemented with 10% fetal bovine serum (FBS,GIBCO) was placed in the lower chamber, and the plate was placed in an CO2 incubator for 48 hours. The cells that migrate to the bottom chamber of cell culture vessels were collected as invasive cell population and were grown in RPMI-1640 medium. This selection process was repeated ten times, and then the cell population from the last selection was validated for invasiveness potential with transwell invasion assay and named A2780-M.

#### 2.1.2. Establishment of Lung Metastasis of A2780-M Cells in Nude Mice

The nude mice were purchased from the Shanghai Stryker Laboratory Animal Co., Ltd. (Shanghai, China), and the animal protocol was approved by experimental animal ethics committee of Zhejiang Cancer Hospital (2016-08-002). A2780-M cells were subcutaneously inoculated into Balb/c nude mice (4 weeks old) at a dose of 75 ×  10^∧^5 cells/ mouse /0.2 mL phosphate buffered saline (PBS). Forty days after inoculation, the nude mice were euthanatized by decapitation followed by autopsy. Lung metastatic tumors were collected and were cut into small pieces. Tumor tissues were then transferred into cell culture flasks and were grown in RMPI-1640 complete medium. 72 hours later, the tumor tissue pieces were removed, and adhered cells were digested with a mixture of 0.5% (w/v) trypsin and 0.02% EDTA (Gibco, CA, USA) at 1:1 (v/v) until half of the cells were detached to fibroblasts. The remaining cells were maintained in cell culture medium for expansion. The process for removing fibroblasts was repeated until no fibroblasts could be observed in culture cells. The tumor cells were then passaged every 3 days, and the cells after twenty passaging were collected as lung metastatic ovarian cancer A2780-M cells.

#### 2.1.3. Short Tandem Repeat (STR) Analysis

Genomic DNAs were extracted from A2780-M and A2780 cells for STR DNA profiling. In brief, the genomic DNA was amplified with PCR reaction, and the converted PCR fragments were analyzed with the ABI (Foster City, CA, USA) 3730XL genetic analyzer to determine STR loci and the sex gene amelogenin.

#### 2.1.4. Cell Proliferation and Cell Cycle Analysis

Log phase growing A2780 and A2780-M cells were collected and plated onto E-Plate VIEW16 (Acea Biosciences Inc., Hangzhou, China) at 4000 cells/well for cell proliferation assay. Cell viability was measured with real-time label-free dynamic cell assays using an EISEN real-time marker-free cell function analyzer (iCELLigence, San Diego, CA, USA).

For cell cycle analysis, cells were plated in 6-well plates at 1x10^∧^5 cells/well and were maintained in serum-free 1640 medium for 24 hours. Cells were then cultured in RPMI-1640 medium containing 10% FBS for additional 24 hours before collection. Collected cells were stained with propidium iodide (PI) and analyzed with flow cytometer (Beckman Coulter CytomicsTM FC 500, Brea, California).

#### 2.1.5. Wound Healing Assay

3x10^∧^4 cells were suspended in 70 uL of RPMI-1640 medium and were seeded in ibidi-Culture-Insert (ibidi-Culture-Insert, Cat. No. 80206; Martinsried, Germany). The incubation chambers were placed in a 5cm diameter cell culture plate. 24 hours later, the insert chamber was removed, and 800 *μ*L serum-free 1640 medium was added to the cell culture plate. Wound Healing analysis was then conducted according to manufacturer's instruction with microscope observation.

#### 2.1.6. Invasion Assay

The incubation chambers were precoated with 1:30 PBE-diluted Matrigel, and 30 *μ*L of 0.1% bovine serum albumin (BSA) solution was added to the chambers before adding 3x10^∧^4 cells suspended in 100 ul of RPMI-1640 medium containing 2% FBS. The incubation chambers were plated on the top of the culture vessels containing 165 *μ*L of RPMI-1640 medium supplemented with 10% FBS. After 30-minute incubation, the cell motility was measured with EISEN real-time marker-free cell function (iCELLigence, San Diego, California, USA)

#### 2.1.7. Chemosensitivity Analysis

4x10^∧^3 cells were cultured in 96-well plates for 24 hours. Cells were then treated with cisplatin (DDP), 7-ethyl-10-hydroxycamptothecin (SN-38), therarubicin (THP), or docetaxel (DTX). The cell viability was determined 48 hours later with cell-Counting kit-8 (CCK8, Dojindo, Japan) according to the manufacturer's instruction.

#### 2.1.8. Flow Cytometry Analysis

Log phase growing A2780 and A2780-M cells were collected and washed with PBS. Cells were then incubated with PE- or FITC-conjugated antibodies for 30 minutes at room temperature. Flow cytometry analysis was used to determine the expression of these cell surface markers, and the data was analyzed with Cellquest analysis software (Beckman CXP). Anti-CD34 antibody was from Jingqiao(Cat: Zhongshan Jinqiao, Beijing, China). Anti-CD133 antibody was from Miltenyi Biotec (Cat: 5151214480, Colner, Germany). Anti-CD117 (Cat: 11996-R007-PE) and anti-CD44 (Cat: 12211-MM02-FITC) antibodies were from Sino Biological Inc. (Beijing, China). Anti-CD24 antibody was from Biolegend (Cat:31105-PE, California,USA)

#### 2.1.9. *β*-Catenin Protein Expression in A2780-M Cells

*β*-Catenin protein expression in A2780-M cells was determined with immunohistochemistry (IHC) and western blot analysis. For IHC, A2780-M cell pellet was embedded in paraffin, and IHC was performed with anti-*β*-catenin (1: 500 dilution, Abcam) antibody and the universal P003IH immunohistochemistry kit (Changsha Yi Jia Biotechnology Co., Ltd, Changshang, Hunan, China). For western blot, A2780-M cells were lysed in Radio Immunoprecipitation Assay Lysis Buffer (RIPA, Beyotime, Jiangsu, China), and 50*μ*g of total protein was subjected for electrophoresis and western blotting. *β*-Actin was included as an internal control.

#### 2.1.10. In Vivo Tumorigenicity

A2780 and A2780-M cells were collected, and 1.2 million cells were inoculated subcutaneously into 4-week-old female SPF BALB/c nude mice (purchased from Shanghai Slack Laboratory Animal Co., Ltd., experimental animal license number SCXK2012-0002). The experimental nude mice were euthanatized after 40 days to determine the tumorigenicity and potential metastasis.

#### 2.1.11. Statistical Analysis

All statistical analyses were carried out using SPSS 13.0 statistical software. Group comparisons were conducted using a* t*-test. P<0.01 was considered statistically significant.

## 3. Results

**Figure 1:**
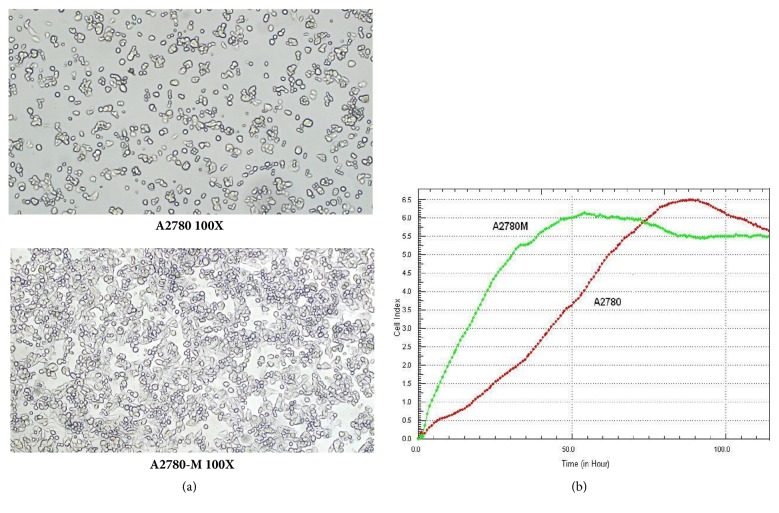
Characterization of A2780-M and A2780 cells. (a) Morphological characteristics of A2780 and A2780-M cells. The A2780 cells are shown to be polygonal or oval in shape (top), and A2780-M cells are more fusiform and fibrous in appearance (bottom); (b) graph shows the different growth rate of A2780-M and A2780 cells.

The morphology examination showed that A2780-M cells appear to be polygonal or oval in shape, which is slightly different from the short fusiform fibers-like morphology of parental A2780 cells (Figure 1). Cell proliferation assay also showed that A2780-M cells grow faster than A2780 cells.

**Figure 2:**
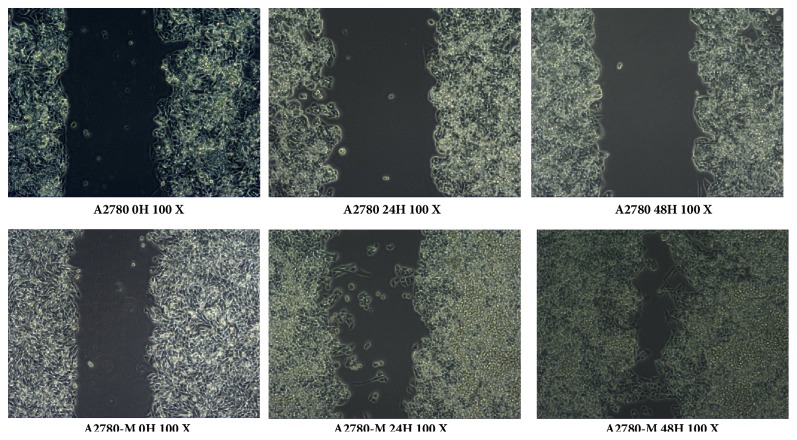
Wound Healing assay representative images showing the difference of cell motility of A2780 (top) and A2780-M cells (bottom) determined with gap refilling analysis.

**Figure 3:**
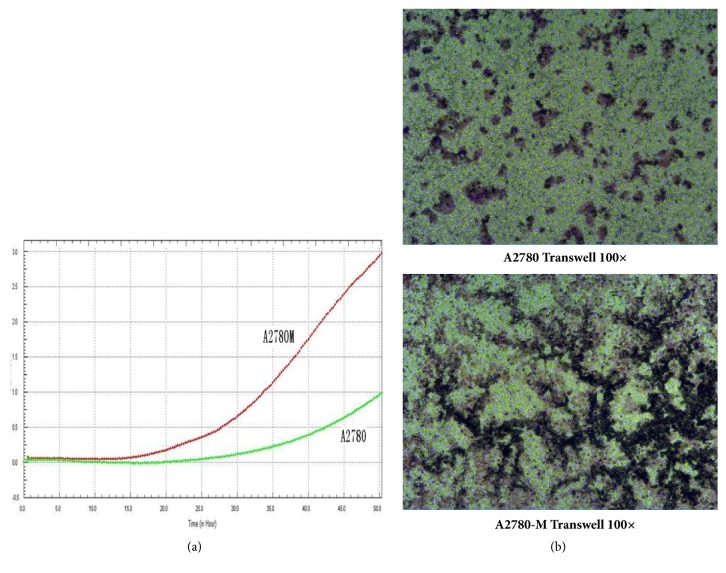
Transwell assay. (a) Graph shows the results of cell motility for A780-M and A2780 cells determined by EISEN real-time marker-free cell function analyzer; (b) representative images showing the results of invaded cells in transwell incubation chambers.

With the STR DNA profiling, we found the DNA genotype of A2780-M cells demonstrated a 100% match with the genomic data of A2780 cells provided in the database of the European Collection of Authenticated Cell Cultures (ECACC) cell bank. The results also revealed the phenomenon of four alleles was not found in any of the individual genes, and no cross-contamination with genome from any known established human cells was observed (Table 1). The cell cycle analysis also showed very similar cell cycling of A2780-M and A2780 cells (Table 2). These results thus indicated that the A2780-M, a single-cell strain, is established from human ovarian A2780 cells. However, the cell motility experiment showed that A2780-M cells could quickly migrate to the middle of the scratch gap area 24 hours after cell seeding, and scratch gap area subsequently disappeared within 48 hours when it was only half filled in the cell culture vessels seeded with A2780 cells (Figure 2). We also observed increased cell numbers of A2780-M cells that penetrated the basement membrane in the electrode-labeled cell-free assay (Figure 3). Thus, the A2780-M cells shows enhanced capabilities of motility invasiveness when compared to the parental A2780 cells.

**Figure 4:**
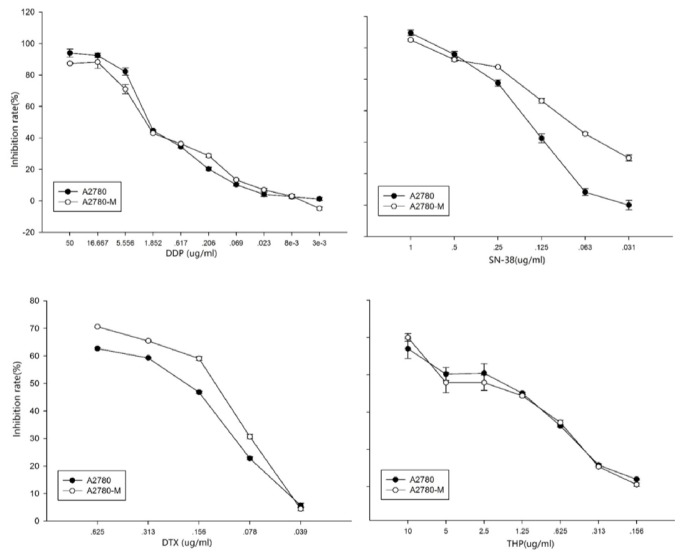
Drug resistance for A2780 and A2780-M. Graphs show the dose responses of A2780-M and A2780 cells to the treatments of DDP (top left), SN-38 (top right), DTX (bottom left), and THP (bottom right). Data represents the average results from at least three independent experiments. Error bars indicate standard deviation.

We next determined the potential differences of A2780 and A2780-M cells in response to the chemodrugs. As shown in Figure 4, we found that A2780-M cells were more resistant to the treatment of SN-38. However, no such difference was observed when cells were exposed to DDP or THP (p>0.05).

**Figure 5:**
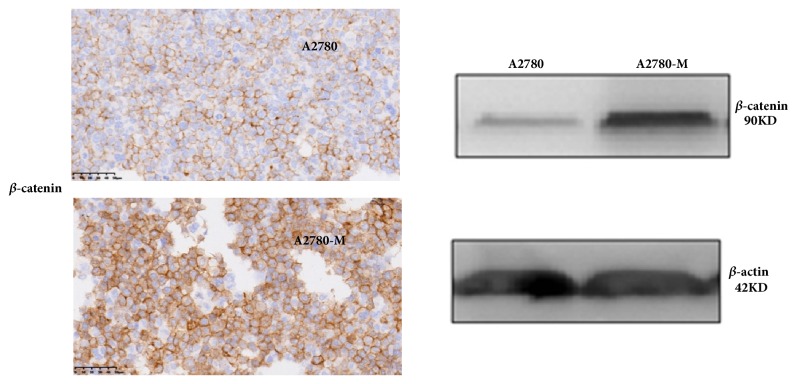
*β*-Catenin expression in A2780 and A2780-M cell lines. (a) Representative images of IHC showing the *β*-catenin expressions; (b) Western blotting results showing the expression of *β*-catenin protein.

With flow cytometry analysis, we detected increased expressions of CD34, CD133, and CD44 in A2780-M cells when compared to parental A2780 cells. No changes were observed for CD24 and CD117 in these two cell lines, however (Table 3). IHC results also showed that while the expression of *β*-catenin in parental A2780 cells was weakly positive and mainly concentrated on the membrane, the expression of *β*-catenin protein appeared to be strongly positive in A2780-M cells and was detected primarily on the cell membrane and in the cytoplasm. In addition, western blotting also validated higher expression of *β*-catenin protein in A2780-M cells (Figure 5).

**Figure 6:**
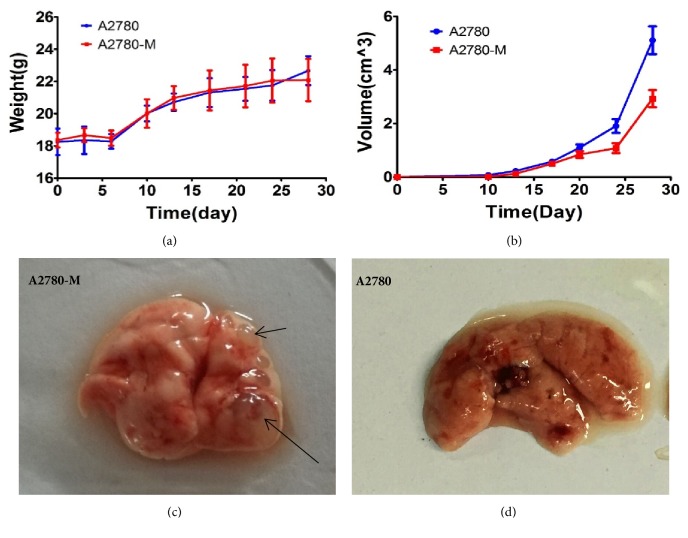
Tumorigenicity analysis. (a) Graph showing the change of body weights for nude mice transplanted with xenografts established from A2780-M and A2780 cells; (b) graph showing the growth rates of xenograft tumors; (c) representative image showing visible lung metastatic nodules in lung organ observed in nude mice bearing A2780-M-xenograft 40 days after tumor cell inoculation; (d) nude mice inoculated with parental A2780 cells showed no metastases in lung organ. Data represents the average results from at least three independent experiments. Error bars indicate standard deviation.

We further determined the tumor growths of xenografts established from A2780 and A2780-M cells. Our results showed that the xenograft tumors established with A2780 cells in nude mice exhibited slightly slower growth than that of A2780 cells, and the tumors of nude mice implanted with A2780 cells were prone to become ulceration. Of interest, we observed increased formation of neovascularization in xenograft tumors of A2780-M cells. We also detected visible lung nodules in the animal inoculated with A2780-M cells. Pathological examination further confirmed lung metastasis in these lung nodules. As expected, no such lung metastasis was observed in the nude mice inoculated with parental A2780 cells (Figure 6).

## 4. Discussion

In this study, we established a lung metastatic cell line, A2780-M, from human ovarian cancer cells, and characterized the biological behaviors of the cell line. Our data demonstrated that A2780-M cell line was a single-cell strain established from human ovarian A2780 cells. Compared to A2780 cells, A2780-M cells exhibit enhanced capabilities of invasiveness and cell motility and the resistances to treatments of SN-38 and DTX. Our results also revealed that A2780-M cells express increased levels of cancer stem cell surface markers such as *β*-catenin, CD34, CD133, and CD44.

*β*-Catenin is a multifunctional protein with dual activities of cell adhesion and signal transduction. *β*-Catenin exists widely in various types of cells such as endothelial cells, fibroblasts, and osteoblasts, and *β*-catenin has been found to play important roles through Wnt signaling pathway in regulations of proliferation, differentiation, and apoptosis of these cells [9]]. The expression of *β*-catenin has also been demonstrated to enhance the stemness and depolarization of tumor stem cells, thus promoting tumor cancer cell invasion and tumor recurrence [10, 11]. For examples, studies have shown that *β*-catenin plays a decisive role in nasopharyngeal carcinoma stem cells through the regulation of the EGFR / AKT gene [12], and overexpression of *β*-catenin has been considered as a biomarker of epithelial–mesenchymal transition (EMT) in prostate stem cells [13]. In addition, *β*-catenin can interact to the DNA binding protein TCF / LEF complex and regulate the expression of C-myc, C-jun, MMP7, and cyclinD and thus affect cell proliferation and metastasis of tumor cells [14–16]. The CD34 molecule was originally found to be a highly glycosylated transmembrane glycoprotein and is selectively expressed on the surface of human hematopoietic stem/progenitor cells. Studies further detected the expression of CD34 in isolated cancer stem cells of leukemia, colorectal cancer, brain tumors, and head and neck cancer, which indicates a potential of CD34 as a surface marker of tumor stem cells. CD34 expression was also found to play important roles in mediating intercellular adhesion and cell migration [17–19]. The data present in this study showed elevated expressions of *β*-catenin and CD34 in A2780-M, suggesting that A2780-M cells are with phenotype of cancer stem cells (CSCs).

CSCs have been demonstrated to be with self-renewal ability and unlimited proliferation, which contribute largely to tumorigenesis and metastasis of cancer cells. The increased capabilities of CSCs for cancer cell invasion and metastasis have been reported in breast cancer, colon cancer, pancreatic cancer, and lung cancer [20–22]. Thus, we propose that the lung metastatic A2780-M cells are initiated and enriched with CSCs of the original A2780 cells. To support this, we also found that A2780-M cells express higher levels of CD133 and CD44, biomarkers for tumor stem cells. CD133 is a transmembrane glycoprotein and a surface marker of hematopoietic stem cells initially found in human embryos, bone marrow, and peripheral blood. Expression of CD133 is involved in the formation and maintenance of different morphologies of cell membrane projections which affects cell polarity, migration of cells, and cell-cell interaction [23, 24]. CD133+ cells show strong mobility capacity and have been demonstrated to contribute to cancer cell invasion and distant metastasis [25, 26]. The CD133 + ovarian cancer stem cells (OCSCs) isolated from ovarian cancer tissue, ascites, and cell lines were found to be capable of initiation of the development and progression of ovarian cancer [27, 28]. CD44 is also important for cell-cell interactions, cell-matrix interactions, cell growth, and differentiation [29]. In gastric and breast cancers, CD44 expression is considered as a solid biomarker of CSCs. [30–32].

In conclusion, the new established A2780-M cells show enhanced capabilities of cell proliferation, cell adhesion, motility and invasion. The STR DNA profiling demonstrated that A2780-M cells share the same genetic background with A2780 cells, suggesting that this cell line can provide a suitable cell model for studying the molecular mechanisms of ovarian cancer metastasis.

## Figures and Tables

**Table 1:** Results of STR typing and DNA genotyping of the A2780-M cells. The results were compared to the database for A2780 cells from the European Collection of Authenticated Cell Cultures (ECACC) cell bank.

Marker	sample				Cellular library information		
	Allele1	Allele2	Allele3	Allele4	Allele1	Allele2	Allele3
D5S818	11	12			11	12	
D13S317	12	13			12	13	
D7S820	10	10			10	10	
D16S539	11	13			11	13	
VWA	15	16			15	16	
TH01	6	6			6	6	
AMEL	X	X			X	X	
TPOX	8	10			8	10	
CSF1PO	10	11			10	11	
D12S391	18	19	20				
FGA	19	24					
D2S1338	21	22					
D21S11	28	28					
D18S51	16	18	19				
D8S1179	15	17					
D3S1358	14	16					
D6S1043	11	19					
PENTAE	10	13					
D19S433	12	12					
PENTAD	9	10					

**Table 2:** Cell cycle analysis for A2780-M and A2780 cells.

Cell	G0-G1 (24H/48H) %	G2-M (24H/48H) %	S (24H/48H) %
A2780	56/58	14.1/12	29.9/30
A2780-M	55/60.5	13/12.5	32/27

**Table 3:** The percentage of cell populations with expressions of cell surface markers CD34, CD24, CD133, CD117, and CD44 in A2780-M and A2780 cells.

Immunophenotype	A2780(%)	A2780-M(%)
CD34-PE	5.6	15.2
CD24-PE	98.3	99.5
CD133-PE	15.9	24.4
CD117-PE	98.8	98.8
CD44-FITC	10.4	27.6

## Data Availability

The data used to support the findings of this study are available from the corresponding author upon request.
